# BPP: a platform for automatic biochemical pathway prediction

**DOI:** 10.1093/bib/bbae355

**Published:** 2024-07-31

**Authors:** Xinhao Yi, Siwei Liu, Yu Wu, Douglas McCloskey, Zaiqiao Meng

**Affiliations:** School of Computing Science, University of Glasgow, 18 Lilybank Gardens, Glasgow G12 8RZ, United Kingdom; Machine Learning Department, Mohamed bin Zayed University of Artificial Intelligence, Building 1B, Masdar City, Abu Dhabi 000000, United Arab Emirates; School of Mathematical Sciences, Fudan University, 220 Handan Rd, Yangpu District, Shanghai 200438, China; Artificial Intelligence, BioMed X Institute, Im Neuenheimer Feld 515, Heidelberg 69120, Germany; School of Computing Science, University of Glasgow, 18 Lilybank Gardens, Glasgow G12 8RZ, United Kingdom

**Keywords:** pathway bioinformatics, biological networks, graph neural networks, hypergraphs

## Abstract

A biochemical pathway consists of a series of interconnected biochemical reactions to accomplish specific life activities. The participating reactants and resultant products of a pathway, including gene fragments, proteins, and small molecules, coalesce to form a complex reaction network. Biochemical pathways play a critical role in the biochemical domain as they can reveal the flow of biochemical reactions in living organisms, making them essential for understanding life processes. Existing studies of biochemical pathway networks are mainly based on experimentation and pathway database analysis methods, which are plagued by substantial cost constraints. Inspired by the success of representation learning approaches in biomedicine, we develop the biochemical pathway prediction (BPP) platform, which is an automatic BPP platform to identify potential links or attributes within biochemical pathway networks. Our BPP platform incorporates a variety of representation learning models, including the latest hypergraph neural networks technology to model biochemical reactions in pathways. In particular, BPP contains the latest biochemical pathway-based datasets and enables the prediction of potential participants or products of biochemical reactions in biochemical pathways. Additionally, BPP is equipped with an SHAP explainer to explain the predicted results and to calculate the contributions of each participating element. We conduct extensive experiments on our collected biochemical pathway dataset to benchmark the effectiveness of all models available on BPP. Furthermore, our detailed case studies based on the chronological pattern of our dataset demonstrate the effectiveness of our platform. Our BPP web portal, source code and datasets are freely accessible at https://github.com/Glasgow-AI4BioMed/BPP.

## Introduction

**Figure 1:**
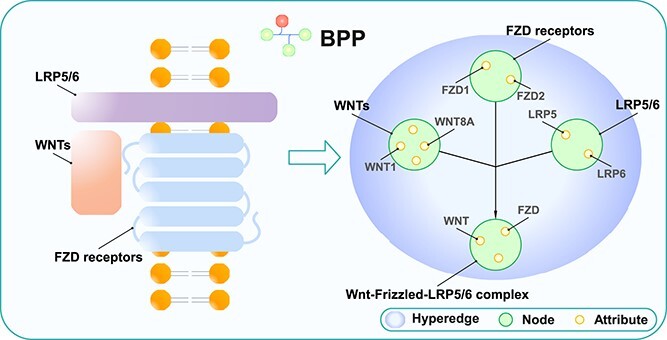
An example showing how to conceptualize a biochemical pathway as a hypergraph, and illustrating graphical representation of link, node, and attribute; the left side depicts the *WNT binding to FZD and LRP5/6 Reaction* within the *WNT signal pathway*, highlighting the interactions among four biochemical entities: *WNTs*, *FZD receptors*, *LRP5/6*, and the resultant *Wnt-Frizzled-LRP5/6 complex*; on the right side, the figure illustrates that BPP represents this *WNT binding to FZD and LRP5/6 Reaction* as a hyperedge, connecting four nodes that represent the biochemical entities involved, and each connection between reaction and entity can be regarded as a link, and by modelling each reaction within the pathway networks as a hyperedge, BPP ultimately represents the entire pathway networks as a hypergraph, and for constructing node attributes, WNTs are regarded as a protein family, encompassing various members such as WNT1 and WNT8A; BPP treats these specific members as attributes of the WNTs; similarly, the Wnt-Frizzled-LRP5/6 complex has its attributes defined by the components WNTs, FZD, and LRP5/6.

Biochemical pathways are a series of reactions involving different types of biochemical entities that occur within a biological system [1]. These pathways are crucial for cellular processes such as disease progression [2–4], metabolism [5, 6], immune system [7, 8], signal transduction [9–11], etc. In biochemical pathways, one reaction typically involves more than two entities such as genes, proteins, and other biomolecules [12], while the products from one reaction often serve as substrates or catalysts for the subsequent reactions [13]. Such a one-to-multiple relation is similar to the spirit of the hypergraph [14], where a hyperedge is usually connected with multiple nodes. Therefore, we can conceptualize a pathway as a hypergraph by considering biochemical entities as nodes and reactions as hyperedges. By representing a pathway as a hypergraph, we establish a natural structure for representing the relationships between reactions and entities, thus simplifying the analysis of biochemical pathways. Figure 1 provides an example of representing the *Wnt binding reaction* within the *Wnt signalling pathway* [15] as a hyperedge while elucidating the instances for constructing node attributes. Notably, biological processes within an organism typically involve multiple pathways, which may intersect and share some common entities. This interconnecting feature leads to the forming of a larger hypergraph, which we refer to as biochemical pathway network. This network serves as an essential framework for representing and analysing intricate networks of interactions among different types of biochemical entities.

One important research task in the field of biochemical pathways is to identify potential links within a pathway [16]. This task has wide applications in identifying novel disease biomarkers [17], discovering new drug targets [18], and addressing gaps of pathway holes in biosynthesis [19, 20]. We focus on identifying potential or missing links between biochemical entities and reactions, which is referred to as the *link prediction* task. This task is crucial for understanding disease pathways, metabolic processes, or cellular responses [21, 22]. Moreover, the entities involved in the link prediction tasks are usually composed of smaller components, e.g. gene fragments in genes and amino acid sequences in proteins. Alternatively, some of these entities can be regarded as a family collection of components. We refer to these components as the *attributes* of entities. These attributes are closely related to entities’ functions in the pathway [23, 24]. As the exploratory nature of experiments involving emerging biochemical entities, certain attributes of these entities may be absent, requiring further investigation [25, 26]. This incompleteness may lead to incomplete structural information within biochemical pathway networks. Therefore, another promising research task is the *attribute prediction* task, i.e. predicting potential or missing attributes of a biochemical entity.

To identify potential links or attributes within biochemical pathway networks, existing methods mainly rely on trial-and-error experimental procedures [1, 27–30]. Due to the vast number of candidates within pathways and the high cost of the experimental process, human experts might devote substantial efforts to identify potential connections through a wide range of experiments and manual analysis. Currently, various databases and platforms integrate pathway knowledge distributed in the literature, providing visual interfaces and search tools [23, 31, 32]. While these resources allow researchers to efficiently conduct experiments and analyse the results based on established pathway knowledge, they still cannot automatically learn the structural information within pathways and make initial predictions of potential connections. In order to further mine structural information within biochemical pathways, this gap highlights an imperative need for methods that can address both link prediction and attribute prediction tasks automatically. To this end, we aim to provide automatic methods to facilitate the identification of potential connections within biochemical pathway networks.

Graph representation learning models provide potential solutions to address the link prediction and attribute prediction tasks automatically [33]. In the context of network data, graph representation learning aims to represent the nodes within a graph as continuous and low-dimensional vectors, which can preserve the topological properties of the original graph [34–36]. Following previous work [37], several methodologies have been proposed for the graph representation learning of network data, such as matrix factorisation (MF) [38], graph neural networks (GNNs) [39], and hypergraph neural networks (HGNNs) [33, 40]. These graph representation learning models have demonstrated success in tackling some biochemical tasks, such as protein–protein interaction prediction [41], gene regulatory prediction [42], and drug–target prediction in metabolic networks [43]. However, their applications in addressing the link prediction and attribute prediction tasks in biochemical pathway networks have not been explored before.

Therefore, we aim to bridge this gap by proposing the Biochemical Pathway Prediction, abbreviated as BPP, an open-source platform for identifying potential connections and attribute characteristics within biochemical pathway networks using various graph representation learning methods, such as MF, GNNs, and HGNNs. BPP primarily focuses on link prediction and attribute prediction tasks. Given the absence of a biochemical pathway network dataset tailored for these two tasks, we build four public datasets based on Reactome [23], one of the most comprehensive pathway databases. We retrieve and process all the human-related pathways available in the Reactome database. These pathways are then classified into different categories based on their domain types. After filtering out categories with limited entities and biochemical reactions, we obtained four distinct datasets: disease, metabolism, immune system, and signal transduction. Each dataset represents a biochemical pathway network in one specific domain. Moreover, BPP incorporates some existing graph representation learning models, such as MF, GNNs, and HGNNs to automatically predict potential connections within biochemical pathway networks. We further employ these models on our four biochemical pathway network datasets to evaluate their performance in link prediction and attribute prediction tasks. Experimental results indicate that these models can offer reliable accuracy in predicting potential connections. Furthermore, given the significance of understanding and explaining pathway predictions, BPP further implements the SHAP explainer [44] that is able to explain the model’s predictive results, i.e. identifying nodes that contribute most to the predictions, enhancing model explainability [44–46]. To the best of our knowledge, BPP is the first platform capable of automatically handling both link prediction and attribute prediction tasks within biochemical pathway networks. Our contributions can be summarized as follows.

- We develop BPP, an open-source biochemical pathway analysis platform dedicated to predicting potential links and node attributes in biochemical pathway networks.
- We evaluate the performance of four graph representation learning models on four biochemical pathway datasets, with experimental results suggesting their consistent and reliable performance of our BPP in the two automated prediction tasks.
- BPP integrates an explainer that provides an explanation of the prediction results, enhancing the prediction explainability of our BPP.
- We verify the effectiveness of BPP by conducting a case study on SARS-CoV-2’s invasion process, which indicates that BPP can successfully identify unseen links within pathways.

The remainder of the paper is organized as follows. First, we introduce three lines of related works: biochemical pathways, graph representation learning and neural network explainer. Then, we detail the architecture of our BPP platform, including the biochemical prediction tasks, the pathway datasets, the graph representation learning models, and the explainer. Subsequently, we outline the experimental settings for evaluating the performance of different graph representation learning models. Next, we provide experimental results and analyses. Finally, we conduct a case study to verify the effectiveness of BPP in successfully predicting potential links within a real-world biochemical pathway network.

## Related work

### Applications for biochemical pathways

Biochemical pathways consist of a chain of reactions where various biochemical entities interact and transform within living organism [1, 12]. The significance of biochemical pathways reside in their vital role in understanding the underlying mechanisms of various biological processes, including disease progression [2–4], metabolism [5, 6], immune system [7, 8], and signal transduction [9–11]. With the integration of computer technology, a variety of applications have emerged in the study of biochemical pathways [32, 47]. Among these, Reactome [23] stands out as a noteworthy public pathway database offering comprehensive pathway information with manually curated molecular details. It enables users to map experimental data, such as gene expression, onto known biological pathways, thus identifying significantly correlated biochemical pathways. Additionally, Pathway Commons [31] serves as an integrated resource platform on biochemical pathways, providing search tools and visualization supported by Systems Biological Graphical Notation. Another notable platform is Pathway Tools [32], which is widely used for visual pathway analysis and specializes in the utilization of various omics data, including genomics and metabolomics. These existing works demonstrate the vast potential applications of pathways while also offering incentive to explore automated methods for biochemical pathways.

### Graph representation learning

Recently, graph representation learning methods have been proposed to facilitate the analyses of graph data [37, 48]. These methods utilize a diverse range of techniques, including MF, GNNs, and HGNNs, to learn the node representations of a given graph, which are subsequently leveraged for downstream predictive tasks, such as link prediction. Specifically, MF proposes to decompose the matrices derived from graph structures, such as adjacency matrices or Laplacian matrices, into low-dimensional matrices that can capture the inherent characteristics of a given graph [33, 38]. Compared with MF, GNNs have gained significant attention due to their superior performance in handling graph-structured data. They update node representations by iteratively aggregating information from neighbouring nodes at each neural network layer [39, 49]. GNNs have also been widely applied in biochemical fields such as genomics, protein structure prediction, and drug discovery [50–52]. For instance, GNNs are used to analyse protein–protein interaction networks, predict gene function, and identify drug targets [53, 54].

In contrast to the MF and GNNs, which learn representations for general graphs, HGNNs aim to specialize GNNs to learn representations in hypergraphs [14]. Specifically, a hypergraph is a graph where an edge can connect more than two nodes, which allows higher-order relationships between entities to be captured. For example, Feng *et al.* [40] proposed a HGNN model that extends GCN to handle hypergraphs through the application of hypergraph Laplacians. HGNN have proven to be effective in a variety of applications, such as classifying citation networks and recognizing visual objects [40]. To further improve the expressive power of HGNN, Gao [55] proposed $\mathrm{HGNN}^{+}$ as a generic HGNN model for learning the representation of hypergraphs. $\mathrm{HGNN}^{+}$ integrates convolutional operations and attention mechanisms to learn information node representations from hypergraphs, which can be applied to a variety of tasks and domains involving hypergraphs. Building upon the existing work in the field, we leverage MF, GNNs, and HGNNs as graph representation learning models for BPP, to learn the structural information of pathways.

### Neural network explainers

Neural network explainers are a crucial category of research tools designed to elucidate and explain the predictions generated by neural network models. The goal of these explainers is to generate explanatory results to help explain the model’s predictions [56, 57]. For example, GNNexplainer [45] identifies the most influential nodes and edges in the subgraph by extracting local subgraphs around the target nodes and training additional explanatory modules. These explanatory results help to understand which parts of the input graph have the greatest impact on the prediction results of the given graph neural network [45]. Parameterised Explainer (P-Explainer) [46] proposes another novel approach to explain the prediction results of GNN models. The main idea of this approach is to introduce an additional parameterised explainer that consists of a neural network for generating the explanatory results [46]. The main difference between GNNexplainer and P-Explainer is that GNNexplainer needs to train a model for each query, while P-Explainer boasts the advantage of building one model for all query cases. Moreover, in order to provide explanations for complex models on large-scale modern datasets, a novel SHAP (Shapley Additive Explanations) framework has been proposed [44]. This framework assigns a specific importance value to each feature for a particular prediction. Its key functionalities include identifying new categories of feature importance measures that unify six existing methods. Moreover, this method demonstrates improved computational performance compared withprevious approaches and is more aligned with human intuition. In BPP, the included prediction explainer is inspired by the principles of the SHAP model. This is because SHAP models can be applied not only to graph neural network models but also to a variety of representation learning models, facilitating future extensions of BPP. This also enables us to analyse the individual contributions of each feature and understand their impacts on the predicted results.

## BPP - A Biochemical Pathway Prediction Platform

**Figure 2:**
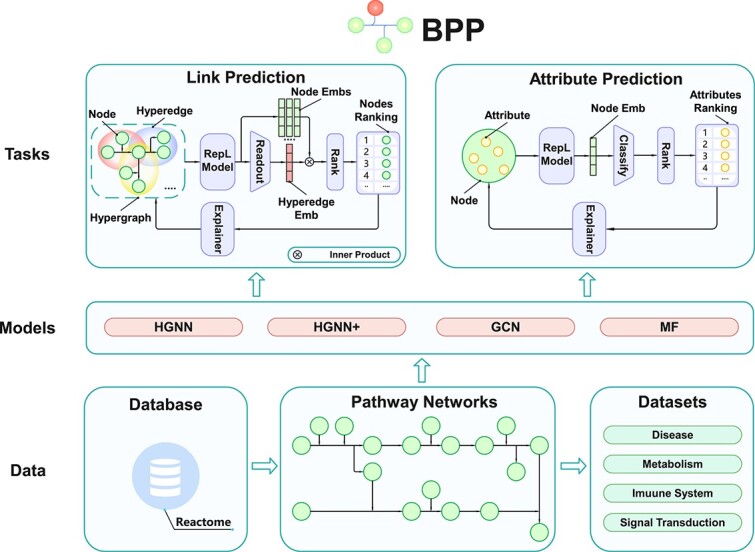
This figure depicts the comprehensive structure of BPP, encompassing two biochemical pathways tasks, four graph representation learning models, and four biochemical pathways datasets; within the illustration, ‘RepL Model’ denotes the representation model within BPP; ‘Readout’ refers to averaging the features of the nodes; ‘Node Emb’ and ‘Hyperedge Emb’ are the abbreviations for node embedding and hyperedge embedding, respectively.

In this section, we introduce the details of our BPP platform. The overall framework of BPP is illustrated in Fig. 2. BPP consists of four main components: the BPP tasks, the datasets tailored for these tasks, different graph representation learning models designed to address these tasks, and an SHAP explainer for explaining the predictions of these models. Specifically, BPP primarily focuses on two BPP tasks, namely the *link prediction* task that predicts potential links in pathway networks and the *attribute prediction* task that unveils latent attributes of entities. Moreover, BPP includes four biochemical pathway datasets, each concentrating on a specific biochemical domain: disease, metabolism, immune system, and signal transduction. These datasets are extracted from Reactome, which is a free, open-source, curated, and peer-reviewed pathway database [23]. In order to address the two pathway prediction tasks automatically, BPP also incorporates several graph representation learning models, which include two HGNN models (HGNN and $\mathrm{HGNN}^{+}$), a GCN model and an MF model. Furthermore, BPP integrates an SHAP explainer to rank the contributions of different entities within the reactions toward the prediction results. Therefore, BPP offers a complete pipeline for biochemical pathway network analysis, which can be used to complement traditional experimental procedures, narrowing down the scope of potential candidates automatically and thereby assisting in pathway research.

### BPP tasks

We begin by introducing the notations used throughout this paper. Let $\mathbf{V}$ denote the set of nodes in the pathway networks, where each node $v \in \mathbf{V}$ represents a biochemical entity, such as a complex, a protein, or a gene. Let $\mathbf{F}$ represent the set of all attributes associated with these biochemical entities, such as molecular components of complexes, amino acid sequences of proteins, and gene fragments of genes. For any $v \in \mathbf{V}$, we use $\mathbf{F}(v) \in \mathbf{F}$ to denote the set of attributes associated with node $v$, with $|\mathbf{F}(v)|$ specifying the number of these attributes. Based on the relationships between entities and their attributes, we represent the features of all nodes as a matrix $\mathbf{X} \in \mathbb{R}^{|\mathbf{V}| \times |\mathbf{F}|}$, where $|\mathbf{V}|$ and $|\mathbf{F}|$ denote the total number of unique nodes and attributes, respectively. Each row of the feature matrix denotes the feature vector of a specific node, with the values chosen from $\{0, 1\}$, where $0$ and $1$ indicate the absence and presence of an attribute within the node, respectively.

In addition, we use $\mathbf{E}$ to denote the set of hyperedges within the pathway networks, where each hyperedge $e \in \mathbf{E}$ represents a biochemical reaction. Given that each hyperedge may involve multiple nodes, we define $\mathbf{V}(e)$ as the set of nodes participating in the hyperedge $e$ and $|\mathbf{V}(e)|$ as the number of nodes within this set. The relationships between all nodes $\mathbf{V}$ and all hyperedges $\mathbf{E}$ within the pathway networks can be effectively represented by an incidence matrix [40] ${\mathbf{H} \in \mathbb{R}^{|\mathbf{V}| \times |\mathbf{E}|}}$, where $|\mathbf{E}|$ denotes the number of hyperedges. The values of the incidence matrix are also chosen from $\{0, 1\}$, indicating the existence of a node within a hyperedge. Moreover, based on the input and output relationships between entities within reactions, we further define an input incidence matrix ${\mathbf{H}_{s} \in \mathbb{R}^{|\mathbf{V}| \times |\mathbf{E}|}}$ and an output incidence matrix ${\mathbf{H}_{t} \in \mathbb{R}^{|\mathbf{V}| \times |\mathbf{E}|}}$, where the values in each column indicate whether an entity serves as the input or output of a specific reaction, respectively. Specifically, the input and output incidence matrix will be leveraged in the input link prediction and output link prediction task, respectively.

Based on the above definition, we denote a biochemical pathway network as $\mathbf{G} = (\mathbf{V}, \mathbf{E}, \mathbf{F}, \mathbf{H}, \mathbf{X})$.

#### Link prediction task

The goal of the link prediction task is to identify the potential links within biochemical pathways. In particular, we identify missing links between biochemical entities and reactions within pathways. Specifically, given a biochemical pathway network $\mathbf{G} = (\mathbf{V}, \mathbf{E}, \mathbf{F}, \mathbf{H}, \mathbf{X})$ and a hyperedge $e$ within the network, the link prediction model within BPP aims to assign a score to each node in the pathway network, which indicates its potential to connect with the given hyperedge $e$, and then rank the nodes that are currently not involved in the hyperedge based on the scores. The top-K nodes are selected as the most promising candidates connecting with the hyperedge.

In a single biochemical reaction, the directional information of the input links between entities and the reaction differs from that of the output links between entities and the reaction. To better leverage the structural information within the biochemical pathway networks and investigate the differences in link directions, we subdivide the link prediction task into *input link prediction* and *output link prediction*. The distinction between these two tasks lies in that the former emphasizes predicting potential input nodes of a reaction, utilizing ${\mathbf{H}_{s} \in \mathbb{R}^{|\mathbf{V}| \times |\mathbf{E}|}}$ as labels during training, while the latter focuses on predicting potential output nodes, exclusively using ${\mathbf{H}_{t} \in \mathbb{R}^{|\mathbf{V}| \times |\mathbf{E}|}}$ as training labels.

#### Attribute prediction task

The goal of the attribute prediction task is to predict the missing attributes of nodes involved in the biochemical reactions. Specifically, given a biochemical pathway network $\mathbf{G} = (\mathbf{V}, \mathbf{E}, \mathbf{F}, \mathbf{H}, \mathbf{X})$ and a node $v$ within the network with known attributes $\mathbf{F}(v)$, the goal of the attribute prediction task is to assign scores for all the attributes within the network, which indicates the probability of these attributes being associated with the node $v$, and rank the attributes that are currently not in $\mathbf{F}(v)$ based on the scores. The top-K attributes are output as the predicted attributes of node $v$.

### Datasets

In order to evaluate the performance of graph representation learning models on the link prediction and attribute prediction tasks, we construct some datasets for BPP based on Reactome [23] that are tailored for these two tasks. To construct these datasets, we first extract all the human-related pathway data from Reactome (version 75) (https://reactome.org/about/news/163-version-75-released). Subsequently, we remove duplicated entities and reactions from these data and categorize these pathway data into multiple categories based on their type labels. These type labels are provided by Reactome, which have been reviewed by experts in the corresponding fields [23]. We then filter out categories with sparse pathway data, i.e. those with a limited number of biochemical entities and reactions. Consequently, this filter-out process yields four distinct pathway categories: disease, metabolism, immune system, and signal transduction. Each category possesses rich pathway data and is vital for in-depth exploration within the biochemical pathway domain. It is worth noting that the models within our BPP platform can also be applied to broader biochemical pathway data.

Therefore, we curate and release four new biochemical pathway datasets tailored for link prediction and attribute prediction tasks within BPP. Each dataset encompasses pathway data corresponding to one of the aforementioned categories. For each dataset, we retain the names of entities, reactions, and attributes. Additionally, we preserve the relationships between reactions and entities, as well as the relationships between entities and their respective attributes within the pathway. Table 1 shows the statistics of these datasets.

**Table 1:** Statistics of our datasets; # RE represents the number of relationships between reactions and entities, and # EA represents the number of relationships between entities and attributes

**Datasets**	**# Reactions**	**# Entities**	**# Attributes**	**# RE**	**# EA**
Disease	1080	1853	2465	3985	4424
Metabolism	2265	2097	2837	9259	3715
Immune System	1623	2643	3413	5389	5880
Signal Transduction	2507	3894	1197	8325	8081

### Models

In order to handle the link prediction and attribute prediction tasks automatically, BPP integrates four graph representation learning models: HGNN, $\mathrm{HGNN}^{+}$, GCN, and MF. Users have the flexibility to select and employ any of these models based on the characteristics of their datasets. In this section, we begin by introducing the pipelines for employing these models to address the link prediction and attribute prediction tasks, respectively. We then proceed to introduce the four graph representation learning models in detail.

#### Pipeline for link prediction

BPP uses a similar pipeline to address both the input link prediction and output link prediction tasks. Given the feature matrix $\mathbf{X}$ and the incidence matrix $\mathbf{H}$ of a pathway network, BPP first uses a graph representation learning model $M$ to learn the embeddings of nodes and then derives hyperedge embeddings based on these node embeddings. To identify potential nodes associated with a hyperedge, it computes the similarities between the embeddings of this hyperedge with the embeddings of all the nodes in the pathway network. Finally, it outputs the top-K most similar nodes as candidates likely to be involved in the reaction. Actually, a higher value of K means that we consider more links predicted by the model as potentially existing. Formally, BPP aims to learn a model $M$ to obtain node embeddings


(1) \begin{align*} \mathbf{X}, \mathbf{H} \xrightarrow{{M}} \mathbf{Z},\end{align*}

where $\mathbf{Z} \in \mathbb{R}^{|\mathbf{V}| \times D}$ denotes the embeddings of all the nodes in the pathway network and $D$ is the dimension of the node embeddings.

Following the hyperedge embedding methods introduced in [58], we apply an average readout function to calculate the hyperedge embeddings, which are the mean embeddings of nodes within the hyperedges. Specifically, the embedding of a hyperedge $e$ within the pathway network can be calculated as


(2) \begin{align*} \mathbf{r}_{e} = \text{Readout}(\mathbf{Z}, \mathbf{G}) = \frac{1}{|\mathbf{V}(e)|} \sum_{v \in \mathbf{V}(e)} \mathbf{z}_{v},\end{align*}

where $\mathbf{r}_{e} \in \mathbb{R}^{D}$ denotes the embedding of the hyperedge $e$ and $\mathbf{z}_{v}$ denotes the embedding of a node $v$ that is involved in the hyperedge, i.e. $\mathbf{V}(e)$. BPP then calculates the inner product of the hyperedge embedding and all the node embeddings


(3) \begin{align*} \boldsymbol{\phi}_{e} = \text{Sigmoid}(\mathbf{r}_{e} \cdot \mathbf{Z}),\end{align*}

where $\text{Sigmoid}(x) = \frac{1}{1 + e^{-x}}$ is an element-wise function and each element of the vector $\boldsymbol{\phi }_{e} \in \mathbb{R}^{|\mathbf{E}|}$ represents the probability that the corresponding node is connected to the hyperedge, i.e., it represents the probability that a biochemical entity is related to the biochemical reaction $e$. To identify potential nodes participating in a particular reaction $e$, we exclude nodes already known to be involved in the reaction and rank the remaining nodes according to their likelihood of being part of the reaction, as determined by $\boldsymbol{\phi }_{e}$.

#### Pipeline for attribute prediction

The procedure employed by BPP to handle the attribute prediction task can be summarized as follows: given the feature matrix $\mathbf{X}$ and the incidence matrix $\mathbf{H}$ of a pathway network, BPP also uses a graph representation learning model $M$ to learn the embeddings of all the nodes in the network. Subsequently, we leverage a linear classifier to predict the probability of nodes having attributes within the network. Finally, for each node, we select the top-K attributes with the highest probability as the potential attributes for the node.

Specifically, similar to the link prediction pipeline, we use a graph representation learning model $M$ to obtain the embeddings of nodes within the network, i.e., $\mathbf{Z}$. Next, BPP employs a linear classifier to estimate the probabilities of node $v$ possessing attributes in the pathway network


(4) \begin{align*}& \boldsymbol{m}_{v} \in \mathbb{R}^{|\mathit{F}|} = \text{Sigmoid} \left( \mathbf{W} \mathbf{z}_{v} + \mathbf{b} \right),\end{align*}

where $\mathbf{W} \in \mathbb{R}^{|\mathbf{F}| \times D}$, $\mathbf{b} \in \mathbb{R}^{|\mathbf{F}|}$ are the parameters of the linear classifier, and each element of the vector $\boldsymbol{m}_{v}$ represents the probability of the corresponding attribute being associated with node $v$. In order to predict the potential attributes of a node $v$, we exclude attributes that are known to be linked with the node and rank the remaining attributes based on their probabilities of being associated with the node, which is determined by $\boldsymbol{m}_{v}$.

#### Graph representation learning models

To better capture the structural information across multiple pathway datasets, BPP incorporates four classic graph representation learning models. Each of these models can be seamlessly integrated into the aforementioned pipelines for both link prediction and attribute prediction. In what follows, we introduce HGNN, $\mathrm{HGNN}^{+}$, GCN, and MF, detailing their application in our pipelines.

- HGNN [40] is a graph neural network model tailored for hypergraphs. It uses an iterative mechanism that enhances node representations by aggregating information from nodes linked via a hyperedge. Simultaneously, it refines the hyperedge representation using information about the nodes [40], adeptly capturing high-order dependencies in hypergraphs. In particular, HGNN learns to update the note embedding following the below formula:
  (5) \begin{align*}& \mathbf{Z}^{(l+1)}=\sigma\left(\mathbf{D}_{v}^{-1 / 2} \mathbf{H} \mathbf{W} \mathbf{D}_{e}^{-1} \mathbf{H}^{\top} \mathbf{D}_{v}^{-1 / 2} \mathbf{Z}^{(l)} \mathbf{\Theta}^{(l)}\right),\end{align*}
  where $\mathbf{Z}^{(l)}$ is the node embeddings at the firth layer of the neural network with $\mathbf{Z}^{(0)} = \mathbf{X}$, $\mathbf{{D}}_{e} \in \mathbb{R}^{|\mathbf{E}| \times |\mathbf{E}|}$ and $\mathbf{{D}}_{v} \in \mathbb{R}^{|\mathbf{V}| \times |\mathbf{V}|}$ are the diagonal matrices denoting the degrees of hyperedges and nodes within the network, respectively, and $\sigma $ is a non-linear activation function. Moreover, $\mathbf{W} = \text{diag}(w_{1}, \dots , w_{|\mathbf{E}|})$ is a diagonal matrix where each value denotes the weight of a hyperedge and $\mathbf{\Theta }^{(l)} \in \mathbb{R}^{D \times D}$ is the feature extractor at the firth layer. Both $\mathbf{W}$ and $\mathbf{\Theta }^{(l)} $ are parameters that are learned during the training process. The HGNN model can be leveraged as the graph representation learning model $M$ in both the link prediction and attribute prediction pipelines. We use the node embeddings at the final layer of HGNN as the output of the model.
-
$\mathrm{HGNN}^{+}$ [55] is an enhanced HGNN model building upon HGNN [40]. It incorporates an adaptive approach to merge distinct hypergraph groups, enhancing the complementarity of information features [55]. The model introduces a two-stage hypergraph convolution rooted in spatial information propagation, ensuring flexibility in both convolution and aggregation operations [55]. The model is formulated as
  (6) \begin{align*}& \mathbf{Z}^{(l+1)}=\sigma\left(\mathbf{D}_{v}^{-1} \mathbf{H} \mathbf{W} \mathbf{D}_{e}^{-1} \mathbf{H}^{\top} \mathbf{Z}^{(l)} \boldsymbol{\Theta}^{(l)}\right).\end{align*}
  The parameters of $\mathrm{HGNN}^{+}$ are similar to that of HGNN. The difference between $\mathrm{HGNN}^{+}$ and HGNN is that $\mathrm{HGNN}^{+}$ defines a discrete and two-stage hypergraph convolution based on message passing from the spatial domain, which is more flexible [55]. Moreover, $\mathrm{HGNN}^{+}$ can also be directly used as the graph representation learning model $M$ in our pipelines.
- GCN [39] is a foundational graph neural network model. In contrast to HGNN and $\mathrm{HGNN}^{+}$, which are proposed for graph representation learning in hypergraphs, GCN is originally proposed for graph representation learning in standard graphs. Specifically, GCN takes the node feature matrix $\mathbf{X}$ and the adjacency matrix $\mathbf{A} \in \mathbb{R}^{|\mathbf{V}| \times |\mathbf{V}|}$ as inputs, where each value in $\mathbf{A}$ is selected from ${0, 1}$ to indicate the absence or presence of a link between two nodes. It then refines a node’s features by incorporating information from its local neighbours. Essentially, GCN amalgamates the graph’s topological data and the node’s features by performing a convolutional operation on the neighbouring nodes, thereby updating the node’s representation. The model’s formulation is given by
  (7) \begin{align*}& \mathbf{Z}^{(l+1)}=\sigma\left(\mathbf{D}^{-\frac{1}{2}} \mathbf{A} \mathbf{D}^{-\frac{1}{2}} \mathbf Z^{(l)} \mathbf{W}^{(l)} \right),\end{align*}
  where $\mathbf{D}$ is a diagonal matrix denoting the degrees of nodes within the network and $\mathbf{W}^{(l)} \in \mathbb{R}^{D \times D}$ denotes the learnable parameters at the firth layer of GCN. In order to apply GCN in our pipelines, we need to transform the pathway network, which is a hypergraph, into a standard graph. Following [59], we utilize $\mathbf{A} = \mathbf{H} \mathbf{H^{\top }}$ to convert the incidence matrix into an adjacency matrix. Essentially, this method transforms a hypergraph into a conventional graph by pairwise connecting all nodes within a hyperedge. We also take the node embeddings at the final layer of GCN as the output of the model.
- MF [60] is one of the most renowned models in the field of graph representation learning. The purpose of introducing the MF model is to evaluate and compare its performance against GNN-based models for handling biochemical pathway data. The MF model’s latent factorization mechanism allows it to capture underlying patterns, making it applicable for predicting entity-reaction relationships. The MF model can be mathematically represented by
  (8) \begin{align*}& \mathbf{H} = \mu\mathbf{Q} + \mathbf{b}_{z} \mathbf{q}_{z}^{\top} + \mathbf{q}_{r} \mathbf{b}_{r}^{\top} + \mathbf{Z}\mathbf{R}^{\top},\end{align*}
  where $\mu $ represents global average rating and $\mathbf{Q} \in \mathbb{R}^{|\mathbf{V}| \times |\mathbf{E}|}$ is a matrix of all ones, $\mathbf{b}_{z} \in \mathbb{R}^{|\mathbf{V}|}$ and $\mathbf{b}_{r} \in \mathbb{R}^{|\mathbf{E}|}$ are learnable parameters representing the bias associated with nodes and hyperedges, respectively, $\mathbf{q}_{z} \in \mathbb{R}^{|\mathbf{E}|}$ and $\mathbf{q}_{r} \in \mathbb{R}^{|\mathbf{V}|}$ are vectors of all ones with different dimensions. Moreover, $\mathbf{Z}$ represents the node embedding matrix, and $\mathbf{R}$ corresponds to the reaction or hyperedge embedding matrix. We use the node embedding matrix $\mathbf{Z}$ as the output of the MF model. Given that the MF model also directly learns a hyperedge embedding matrix $\mathbf{R}$, we use this matrix to calculate the predicted incidence matrix during the link prediction task.

### SHAP explainer for BPP

Once we obtain the predicted results, a natural question arises: how are these predictions generated, i.e. which nodes contribute to the predictions [45]. Taking the example of predicting product links in biochemical reactions, it is important to identify the participating entities or existing products that contribute significantly to the predicted results. This allows researchers to examine the plausibility of the predictions from a biochemical perspective and enhances the transparency and clarity of the entire prediction process. SHAP value is a widely used metric for measuring the importance of input features to predictions [44]. The central idea of the SHAP value is to exclude a feature from the inputs and calculate the difference in model predictions as the significance score of the feature. Specifically, it measures the importance of a feature to model prediction by calculating the following formula:


(9) \begin{align*}& \phi_{i}(x) = \sum_{S \subseteq N \setminus \{i\}} \frac{|S|!(|N|-|S|-1)!}{|N|!} [f(x_{S \cup \{i\}}) - f(x_{S})],\end{align*}

where $N$ is the set of all features, and $S$ denotes all the possible subsets of features excluding feature $i$ and $f$ denotes a model. The $f(x_{S \cup \{i\}})$ denotes the predicted output of the model after adding the feature $i$ to the subset $S$, and $f(x_{S})$ denotes the predicted output of the model when only the features of the subset $S$ are used.

We leverage the SHAP values of nodes and attributes to measure their importance on the prediction results for a given reaction. Specifically, we treat a node or an attribute in the pathway network as feature $i$ in Equation (9) and perform node masking and attribute masking to obtain the subset $S$. These masking methods systematically generate input examples by selectively removing nodes and attributes. Specifically, we mask the nodes by exhaustively considering all possible combinations of masked nodes until we obtain samples for all possible cases. Next, we perform the same procedure for masking attributes until we have obtained all the samples. Moreover, we set an upper limit during the process of generating samples to prevent an excessive number of samples due to too many nodes or attributes. Once the SHAP values for all the nodes and the attributes have been calculated, we rank them according to their SHAP values, with the top-ranked nodes and attributes being considered as the ones that have the biggest impact on model predictions.

## Experimental settings

### Data splitting

We use two different data-splitting methods, namely the random split and temporal split. In Random splitting, we randomly mask certain associations within the pathways and leverage the models in our BPP to predict these masked associations, thereby enabling us to assess and compare the performance of each benchmark. For tasks such as link prediction and attribute prediction, we split the data into training, validation and test sets in an 8:1:1 distribution. Using link prediction as an illustrative case, we randomly select the biochemical reactions. Then, we mask the relationship between the reaction and an input entity for input link prediction or the relationship between the reaction and an output entity for output link prediction. These masked relationships are utilized for validation and testing purposes. Finally, we ensure that the proportion of relationships across the training, validation, and test sets adheres to the 8:1:1 ratio.

**Figure 3:**
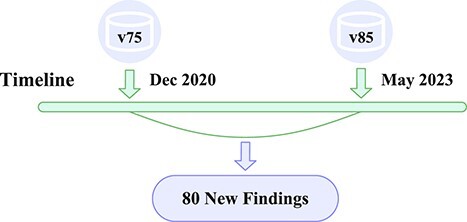
Compare data from 2020 with data from 2023, using the set of differences between the different temporal datasets as the set of newly discovered links.

Temporal splitting is employed to evaluate BPP’s predictive capability on newly identified links. We extract the latest dataset based on the Reactome version 85 dataset (released in May 2023) (https://reactome.org/about/news/225-v85-released) and compare it with the version 75 dataset (released in December 2020) that we use for simulate the real-world scenario. By comparing these two versions, we identify 80 new links in the version 85 dataset as unseen links. In order to evaluate the ability of our models to predict potential links that are not present in the current dataset, we train our model on the version 75 datasets and evaluate the models’ performance on the 80 new links (see Fig. 3 for a timeline illustration for these two datasets).

### Evaluation metrics

Considering that the final prediction result is a ranking of potential links, we use NDCG [61] and Accuracy [62] at a cut-off K (i.e. NDCG@K and ACC@K) to evaluate the performance. NDCG@K is a widely used evaluation metric in the field of information retrieval and recommender systems to assess the quality of ranking, where K indicates that only the first K items in the ranked list are taken into account[63]. In terms of ACC@K, a prediction is considered to be accurate if the correct item appears in the top-K predicted items. It assesses how well the model performs in terms of providing accurate and relevant results within the specified cutoff of K. Therefore, we set various cut-offs to assess the performance of the metrics at different levels of difficulty. Notably, due to space constraints, we only give the results with $K=1$ and $K=10$. Moreover, all the links among the top K predictions, apart from the true missing links, are considered false positives.

### Implementation details

The experimental pipeline for BPP is constructed using PyTorch, a widely recognized deep learning framework. For both link prediction and attribute prediction tasks, we employ the cross-entropy loss as our loss function, which is defined as follows:


(10) \begin{align*}& H(p, q) = -\sum_{i} p(i) \log q(i).\end{align*}

The rationale behind this selection is the efficacy of cross-entropy in measuring the difference between the actual and predicted probabilities. To update the parameters, we use Adam [64] as the optimiser. We set up a 5-fold cross-validation to make our results more robust. Specifically, the standard deviation of the cross-validation is shown in parentheses for individual data. We set the dimension of the hidden layer from [64, 128, 256] and select the learning rate from [0.05, 0.01, 0.005] based on the performance of the validation sets. Please refer to Table 6 for the specific hyperparameter settings of the experiment.

## Results and analysis

This section presents experimental results for the link prediction task, the attribute prediction task, and the performance of real discoveries obtained based on time splitting. The results of the SHAP explainer will be analysed in the case study section.

### Link prediction

We assess the performance of various models across different datasets to help users select the most effective models for their specific needs within BPP. The results of different graph representation learning models in the input link prediction and output link prediction tasks are shown in Tables 2 and 3, respectively. In the task of input link prediction, some very close results indicate that these models have similar predictive abilities on those datasets. For example, in Table 2, under Signal Transduction NDCG, the HGNN scores 0.2975, while GCN scores 0.2947; similarly, in ACC, HGNN scores 0.0969 and GCN 0.0947. This suggests that the hypergraph-based HGNN and the conventional graph-based GCN have comparable predictive capabilities on the Signal Transduction dataset, with HGNN slightly outperforming GCN by a narrow margin. A possible reason is that the Signal Transduction dataset features an abundant set of relationships, totalling 8325, which implies that the dataset is abundant in first-order relational information. Consequently, GCN that can effectively learn first-order relational information tends to perform well. Correspondingly, for the Disease dataset, the number of relationships is more limited, totalling 3985. This indicates that the dataset contains relatively less first-order relational information. Therefore, as seen in Tables 2 and 3, the hypergraph-based HGNN, which is highly beneficial for capturing sparse and high-order information, generally outperforms the GCN. In the output link prediction task, the prediction performance of the different models is consistently compared with the prediction results of the input link prediction task. In the disease datasets, the overall performance of HGNN remains superior to that of GCN. However, on the immune system dataset, the situation is different, and MF outperforms the other three models, which we believe is due to the fact that for the immune system, its output links tend not to have too many complex multi-hop linking relationships, and thus, the basic MF model also learns the structural information well.

**Table 2:** Final results of input link prediction; notably, all the test datasets contain more than 1.8 K biochemical entities, and specifically, in the signal transduction dataset, BPP is required to identify the most relevant candidate among 3894 biochemical entities

**Datasets**	**Models**	**NDCG**	**NDCG@10**	**ACC**	**ACC@10**
**Disease**	**HGNN**	**0.2959 ($\pm $ 0.0122)**	**0.0134 ($\pm $ 0.0163)**	**0.0941 ($\pm $ 0.0185)**	**0.3465 ($\pm $ 0.0144)**
	$\mathbf{HGNN}^{+}$	0.2702 ($\pm $ 0.0158)	0.1662 ($\pm $ 0.0110)	0.0693 ($\pm $ 0.0187)	0.2871 ($\pm $ 0.0139)
	**GCN**	0.2931 ($\pm $ 0.0139)	0.1899 ($\pm $ 0.0120)	0.0891 ($\pm $ 0.0175)	0.3218 ($\pm $ 0.0103)
	**MF**	0.2651 ($\pm $ 0.0109)	0.1296 ($\pm $ 0.0138)	0.0495 ($\pm $ 0.0174)	0.2277 ($\pm $ 0.0125)
**Metabolism**	**HGNN**	0.2734 ($\pm $ 0.0134)	0.1756 ($\pm $ 0.0154)	0.0870 ($\pm $ 0.0146)	0.2826 ($\pm $ 0.0157)
	$\mathbf{HGNN}^{+}$	0.2381 ($\pm $ 0.0107)	0.1378 ($\pm $ 0.0134)	0.0674 ($\pm $ 0.0196)	0.2261 ($\pm $ 0.0113)
	**GCN**	**0.2859 ($\pm $ 0.0150)**	**0.1836 ($\pm $ 0.0103)**	**0.1043 ($\pm $ 0.0149)**	**0.3022 ($\pm $ 0.0106)**
	**MF**	0.2612 ($\pm $ 0.0117)	0.1381 ($\pm $ 0.0155)	0.0500 ($\pm $ 0.0145)	0.2543 ($\pm $ 0.0107)
**Immune System**	**HGNN**	0.3407 ($\pm $ 0.0147)	0.2533 ($\pm $ 0.0156)	**0.1199 ($\pm $ 0.0179)**	0.4212 ($\pm $ 0.0117)
	$\mathbf{HGNN}^{+}$	0.2968 ($\pm $ 0.0119)	0.1976 ($\pm $ 0.0138)	0.0651 ($\pm $ 0.0155)	0.3562 ($\pm $ 0.0135)
	**GCN**	**0.3429 ($\pm $ 0.0149)**	**0.2615 ($\pm $ 0.0134)**	0.1062 ($\pm $ 0.0183)	**0.4623 ($\pm $ 0.0141)**
	**MF**	0.2327 ($\pm $ 0.0115)	0.1035 ($\pm $ 0.0165)	0.0377 ($\pm $ 0.0148)	0.1815 ($\pm $ 0.0141)
**Signal Transduction**	**HGNN**	**0.2975 ($\pm $ 0.0141)**	**0.2042 ($\pm $ 0.0133)**	0.0859 ($\pm $ 0.0157)	0.3436 ($\pm $ 0.0170)
	$\mathbf{HGNN}^{+}$	0.2484 ($\pm $ 0.0103)	0.1429 ($\pm $ 0.0134)	0.0617 ($\pm $ 0.0188)	0.2511 ($\pm $ 0.0148)
	**GCN**	0.2947 ($\pm $ 0.0117)	0.1957 ($\pm $ 0.0135)	**0.0947 ($\pm $ 0.0173)**	**0.3480 ($\pm $ 0.0144)**
	**MF**	0.2198 ($\pm $ 0.0122)	0.0892 ($\pm $ 0.0168)	0.0286 ($\pm $ 0.0193)	0.1608 ($\pm $ 0.0144)

**Table 3:** Final results of output link prediction

**Datasets**	**Models**	**NDCG**	**NDCG@10**	**ACC**	**ACC@10**
**Disease**	**HGNN**	**0.3645 ($\pm $ 0.0101)**	**0.2925 ($\pm $ 0.0102)**	0.1616 ($\pm $ 0.0183)	**0.4646 ($\pm $ 0.0104)**
	$\mathbf{HGNN}^{+}$	0.2865 ($\pm $ 0.0105)	0.1905 ($\pm $ 0.0106)	0.0808 ($\pm $ 0.0177)	0.3434 ($\pm $ 0.0108)
	**GCN**	0.3643 ($\pm $ 0.0110)	0.2646 ($\pm $ 0.0112)	**0.1717 ($\pm $ 0.0163)**	0.4545 ($\pm $ 0.0114)
	**MF**	0.3631 ($\pm $ 0.0115)	0.2877 ($\pm $ 0.0116)	0.1414 ($\pm $ 0.0158)	0.4242 ($\pm $ 0.0124)
**Metabolism**	**HGNN**	0.2927 ($\pm $ 0.0126)	0.1987 ($\pm $ 0.0127)	0.0851 ($\pm $ 0.0178)	0.3252 ($\pm $ 0.0130)
	$\mathbf{HGNN}^{+}$	0.2459 ($\pm $ 0.0131)	0.1421 ($\pm $ 0.0132)	0.0578 ($\pm $ 0.0163)	0.2553 ($\pm $ 0.0134)
	**GCN**	**0.3174 ($\pm $ 0.0136)**	**0.2212 ($\pm $ 0.0137)**	**0.1155 ($\pm $ 0.0188)**	**0.3495 ($\pm $ 0.0139)**
	**MF**	0.2701 ($\pm $ 0.0140)	0.1459 ($\pm $ 0.0141)	0.0608 ($\pm $ 0.0172)	0.2948 ($\pm $ 0.0144)
**Immune System**	**HGNN**	0.2562 ($\pm $ 0.0145)	0.1502 ($\pm $ 0.0146)	0.0813 ($\pm $ 0.0147)	0.2846 ($\pm $ 0.0148)
	$\mathbf{HGNN}^{+}$	0.2070 ($\pm $ 0.0149)	0.1020 ($\pm $ 0.0150)	0.0244 ($\pm $ 0.0151)	0.2276 ($\pm $ 0.0152)
	**GCN**	0.2496 ($\pm $ 0.0154)	0.1447 ($\pm $ 0.0156)	0.0732 ($\pm $ 0.0160)	0.2520 ($\pm $ 0.0166)
	**MF**	**0.3352 ($\pm $ 0.0146)**	**0.2071 ($\pm $ 0.0148)**	**0.0976 ($\pm $ 0.0149)**	**0.3415 ($\pm $ 0.0101)**
**Signal Transduction**	**HGNN**	0.2458 ($\pm $ 0.0147)	0.1341 ($\pm $ 0.0103)	0.0819 ($\pm $ 0.0163)	0.2281 ($\pm $ 0.0113)
	$\mathbf{HGNN}^{+}$	0.2353 ($\pm $ 0.0148)	0.1345 ($\pm $ 0.0145)	0.0818 ($\pm $ 0.0147)	0.2105 ($\pm $ 0.0107)
	**GCN**	**0.2600 ($\pm $ 0.0142)**	**0.1538 ($\pm $ 0.0134)**	**0.0877 ($\pm $ 0.0193)**	**0.2515 ($\pm $ 0.0103)**
	**MF**	0.2430 ($\pm $ 0.0134)	0.1418 ($\pm $ 0.0128)	0.0702 ($\pm $ 0.0164)	0.2456 ($\pm $ 0.0103)

### Attribute prediction

The results of different graph representation learning models on the attribute prediction task are shown in Table 4. Specifically, HGNN+ consistently outperforms other models across all four datasets. While its performance in link prediction may not be its strongest suit, it undeniably stands out as an optimal graph representation learning model tailored for the attribute prediction task within the BPP framework. On the other hand, the MF approach appears to lag considerably in attribute prediction. This could be due to its inherent design, which seems to be more geared toward discerning relationships between nodes and attributes and does not fully harness the comprehensive structural information embedded within the entire pathway networks. The performance of both HGNN and GCN in the attribute prediction, although commendable, still falls short when compared with HGNN+. It is worth mentioning that both HGNN and GCN exhibit respectable results not just in attribute prediction but also in link prediction. This dual competency makes them viable contenders if one seeks a unified model adept at handling both tasks. In scenarios where an integrated approach for both link and attribute prediction is prioritized, both HGNN and GCN emerge as plausible selections, balancing effectiveness across tasks.

**Table 4:** Final results of attribute prediction

**Datasets**	**Models**	**NDCG**	**NDCG@10**	**ACC**	**ACC@10**
**Disease**	**HGNN**	0.4121 ($\pm $ 0.0096)	0.3517 ($\pm $ 0.0125)	0.2153 ($\pm $ 0.0150)	0.5127 ($\pm $ 0.0115)
	$\mathbf{HGNN}^{+}$	**0.4212** **($\pm $ 0.0153)**	**0.3620** **($\pm $ 0.0120)**	**0.2323** **($\pm $ 0.0144)**	**0.5184** **($\pm $ 0.0104)**
	**GCN**	0.4134 ($\pm $ 0.0123)	0.3518 ($\pm $ 0.0101)	0.2210 ($\pm $ 0.0157)	0.5014 ($\pm $ 0.0168)
	**MF**	0.3918 ($\pm $ 0.0158)	0.3325 ($\pm $ 0.0112)	0.1987 ($\pm $ 0.0119)	0.4832 ($\pm $ 0.0185)
**Metabolism**	**HGNN**	0.3377 ($\pm $ 0.0166)	0.2576 ($\pm $ 0.0131)	0.1441 ($\pm $ 0.0176)	0.4279 ($\pm $ 0.0100)
	$\mathbf{HGNN}^{+}$	**0.3397** **($\pm $ 0.0176)**	**0.2663** **($\pm $ 0.0187)**	**0.1577** **($\pm $ 0.0147)**	**0.4279** **($\pm $ 0.0165)**
	**GCN**	0.3383 ($\pm $ 0.0157)	0.2661 ($\pm $ 0.0122)	0.1532 ($\pm $ 0.0184)	0.4099 ($\pm $ 0.0160)
	**MF**	0.3212 ($\pm $ 0.0093)	0.2542 ($\pm $ 0.0179)	0.1428 ($\pm $ 0.0177)	0.4062 ($\pm $ 0.0171)
**Immune System**	**HGNN**	0.3062 ($\pm $ 0.0149)	0.2306 ($\pm $ 0.0092)	0.1139 ($\pm $ 0.0163)	0.3656 ($\pm $ 0.0114)
	$\mathbf{HGNN}^{+}$	**0.3142** **($\pm $ 0.0136)**	**0.2416** **($\pm $ 0.0109)**	**0.1224** **($\pm $ 0.0190)**	**0.3724** **($\pm $ 0.0107)**
	**GCN**	0.3108 ($\pm $ 0.0130)	0.2352 ($\pm $ 0.0117)	0.1293 ($\pm $ 0.0146)	0.3622 ($\pm $ 0.0134)
	**MF**	0.2988 ($\pm $ 0.0173)	0.2212 ($\pm $ 0.0095)	0.0984 ($\pm $ 0.0180)	0.3514 ($\pm $ 0.0141)
**Signal Transduction**	**HGNN**	0.3376 ($\pm $ 0.0155)	0.2664 ($\pm $ 0.0138)	0.1337 ($\pm $ 0.0139)	0.4245 ($\pm $ 0.0128)
	$\mathbf{HGNN}^{+}$	**0.3572** **($\pm $ 0.0090)**	**0.2900** **($\pm $ 0.0169)**	**0.1547** **($\pm $ 0.0174)**	**0.4443** **($\pm $ 0.0152)**
	**GCN**	0.3454 ($\pm $ 0.0188)	0.2758 ($\pm $ 0.0182)	0.1374 ($\pm $ 0.0142)	0.4356 ($\pm $ 0.0103)
	**MF**	0.3126 ($\pm $ 0.0111)	0.2457 ($\pm $ 0.0133)	0.1104 ($\pm $ 0.0161)	0.4156 ($\pm $ 0.0098)

### Real-world temporal split

As previously mentioned under the ‘temporal splitting’ part, we compare datasets from different time periods to collate links that have been newly identified within a specific period in the real world. These links stand in contrast to the ones we manually masked in our initial test set. Being genuine recent discoveries, these links offer greater validation value, serving as a more authentic benchmark for evaluation. From the final results in Table 5, it can be observed that both HGNN and GCN demonstrate promising performance on the newly identified links, as indicated by the ACC@10 metrics. Despite the relatively small sample size, these results provide evidence that our trained models possess a certain level of credibility when transferred to real-world scenarios.

**Table 5:** Performance results based on datasets obtained through temporal splitting, covering two specific time points: December 2020 and May 2023; in this process, we identify 80 new findings and verify the performance of BPP on them, and this is done to evaluate the effectiveness of BPP in predicting real-world potential connections within pathway networks

**Models**	**NDCG**	**NDCG@10**	**ACC**	**ACC@10**
**HGNN**	0.3027	0.1920	**0.0750**	0.3625
** $\mathbf{HGNN}^{+}$ **	0.1505	0.0258	0.0125	0.0375
**GCN**	**0.3135**	**0.2014**	0.0500	**0.3875**
**MF**	0.2374	0.1280	0.0250	0.2625

**Table 6:** Hyperparameters for experimental setup

**Hyperparameters**	**GNN**			**MF**			
	**Emb Dim**	**LR**	**Epoch**	**Emb Dim**	**LR**	**Epoch**	**Batch Size**
**Disease**	256	5e-3	200	256	5e-3	200	64
**Metabolism**	256	5e-3	200	256	5e-3	200	128
**Immune System**	256	5e-3	200	256	5e-3	200	128
**Signal Transduction**	128	5e-3	200	256	5e-3	200	64

Although it is essential to consider the limitations of a small dataset, the positive outcomes achieved by our trained models on the Metabolism dataset provide promising prospects for their applicability in real-world scenarios. These results encourage further exploration and validation of the models on larger datasets and in practical biochemical settings. Moreover, the results of real-world temporal splitting also indicate that false positives predicted by the model are also of great importance, as they may be real links that are not known to humans at the moment.

In summary, the encouraging performance of HGNN and GCN, as reflected by the ACC@10 metrics on the Metabolism dataset, supports the credibility and potential applicability of our trained models in real-world scenarios. These findings lay the foundation for future research and practical applications in the field of biochemical pathways.

## Case study

**Figure 4:**
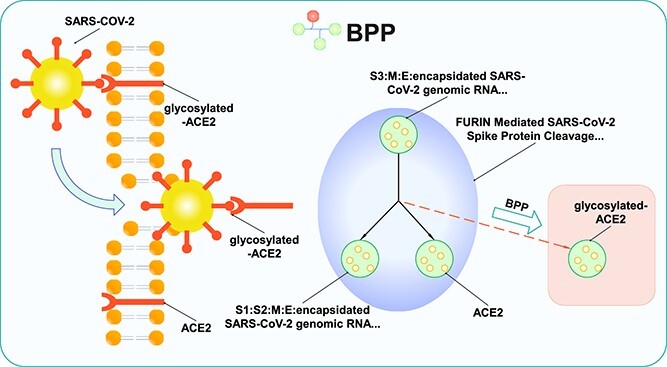
Case Study: Unveiling *Glycosylated-ACE* as a Potential receptor for *SARS-CoV-2* viral entry into host cells; with the benefit of BPP, we identify a link between the reaction *FURIN Mediated SARS-CoV-2 Spike Protein Cleavage and Endocytosis* and the biochemical entity *glycosylated-ACE2*, which is unseen in the original biochemical pathway networks, and these findings align with recent studies suggesting *glycosylated-ACE*’s role as a potential receptor for *SARS-CoV-2*’s invasion into cells.

This case study, illustrated in Figure 4, aims to demonstrate the predictive ability of BPP in link prediction and how the explainer provides explanations of the outputs. Specifically, we train and evaluate the link prediction models within BPP on the disease dataset. We find that BPP can successfully identify a connection between a protein receptor (node) *glycosylated-ACE2* and a biochemical reaction (hyperedge) *FURIN Mediated SARS-CoV-2 Spike Protein Cleavage and Endocytosis*. This reaction is a crucial step in *SARS-CoV-2*’s invasion into host cells [65, 66] and will be referred to as *cleavage reaction* for brevity in the following text. Indeed, within the disease dataset in BPP, which is constructed from Reactome (version 75), there is no direct connection between *glycosylated-ACE2* and the *cleavage reaction*, i.e., no linkage between the corresponding node and hyperedge in the pathway network. However, recent scientific researches have revealed that *glycosylated-ACE2* can act as a receptor involved in *cleavage reaction*, which plays an important role in *SARS-CoV-2*’s invasion [67–69]. Moreover, the connection between *glycosylated-ACE2* and *cleavage reaction* is also recorded in the later version of Reactome (version 85). These findings suggest that there exists a link between *glycosylated-ACE2* and *cleavage reaction*. In our experiment, we find that BPP can successfully rank *glycosylated-ACE2* within the top-$10$ out of roughly 1853 candidates in the disease dataset. This result indicates the effectiveness of BPP in predicting potential connections between biochemical entities and reactions within real-world biochemical pathway networks.

Subsequently, we examine the effectiveness of BPP’s SHAP explainer in explaining the predicted interaction between *glycosylated-ACE2* and the *cleavage reaction*. Specifically, the SHAP explainer generates weights for each node and its attributes in the *cleavage reaction*, which represents the significance of these nodes or attributes to the model’s predicted outcome. The results of the SHAP explainer are provided in Table 7. It shows that *ACE2* ranks first among all the nodes and attributes, which suggests that *ACE2* plays the most significant role in this prediction. Such an explanation is reasonable since the link prediction model may leverage the similarity between *ACE2* and *glycosylated-ACE2* to infer the missing link. The *encapsidated SARS-CoV-2 genomic RNA*, *S1:S2:M lattice:E prote*, and *7a:O-glycosyl 3a tetrame* rank second, third, and fourth, respectively. Indeed, all of them are the attributes of the same node *S1:S2:M:E:encapsidated SARS-CoV-2 genomic RNA: 7a:O-glycosyl 3a tetramer*. Considering that this node represents the spike protein complex, which has a direct binding affinity with glycosylated-ACE2, it is reasonable that these attributes, as components of the spike protein complex, play a significant role in predicting the connection between *glycosylated-ACE2* and the *cleavage reaction*. To conclude, our SHAP explainer demonstrates reliable explainability and rationale in this case study, indicating its effectiveness as a component within BPP.

**Table 7:** Results of SHAP explainer for the predicted interaction between *glycosylated-ACE2* and the *cleavage reaction*, where Attr. denotes attributes; the nodes and attributes are ranked based on their significance to the prediction

**Name**	**Ranking**
ACE2 (Node)	1
encapsidated SARS-CoV-2 genomic RNA (Attr.)	2
S1:S2:M lattice:E protein (Attr.)	3
7a:O-glycosyl 3a tetramer (Attr.)	4
S3:M:E:encapsidated SARS-CoV-2 genomic RNA: 7a:O-glycosyl 3a tetramer:glycosylated-ACE2 (Node)	5

## Conclusion

In this paper, we introduce the BPP platform, designed specifically for predicting links and attributes in biochemical pathways. We validate BPP’s effectiveness through comprehensive evaluations across four diverse datasets, demonstrating its capability for both link and attribute prediction. Notably, we implement an SHAP explainer to assess the contribution of each participant, enhancing our understanding of model prediction. A distinguished achievement of our work is the successful prediction of a key receptor, glycosylated-ACE2, instrumental in the SARS-CoV-2 invasion process. This discovery proffers invaluable insights for a deeper understanding and possible containment strategies for such viruses. In essence, BPP offers an open-source tool for biochemical pathway research, holding the potential to make impacts in the realm of life sciences.

**Key Points**

- We build BPP, an open-source platform for analysing biochemical pathways, focused on predicting links and node attributes within pathway networks.
- The evaluation of four graph representation learning models across four distinct biochemical pathway datasets confirms BPP’s reliable performance.
- We integrate an SHAP explainer in BPP to enhance the explainability of predictions.
- A case study on the SARS-CoV-2 invasion process validates BPP’s effectiveness in identifying undiscovered connections within pathways.

## Data Availability

All data and codes have been released on GitHub.
